# Celebrating 1 year of *EHJ-IMP*: imaging an open world

**DOI:** 10.1093/ehjimp/qyae031

**Published:** 2024-05-01

**Authors:** Alessia Gimelli, Gerald Maurer

**Affiliations:** Imaging Department, Fondazione Toscana Gabriele Monasterio, Via Moruzzi 1, 56124 Pisa, Italy; Division of Cardiology, Department of Medicine II, Medical University of Vienna, Wien, Austria

As we mark the completion of our first year online, the *European Heart Journal—Imaging Methods and Practice* (*EHJ-IMP*) takes pride in its mission to visualize an open world where expertise converges to enhance patient care. Founded on principles of inclusivity, innovation, and collaboration, *EHJ-IMP* would like to facilitate the exchange of cutting-edge research, insights, and experiences in the cardiological community.

At *EHJ-IMP*, we have guiding principles that shape our endeavour: (i) Facilitating dissemination: Through open access, we strive to bridge the gap between ground-breaking research and its accessibility to a wide readership in the field of cardiology. (ii) Inclusivity: *EHJ-IMP* embraces diverse perspectives and contributions from all corners of the cardiovascular imaging community from technological advancements to clinical breakthroughs. (iii) Stimulating communication: Through scientific updates and educational resources *EHJ-IMP* serves as a hub for collaboration, fostering interdisciplinary exchanges that drive progress in patient care.

One year on from the first publication, we would like to highlight and celebrate some of the most important studies that were published in the first 12 months of the Journal.

## Original articles

Cremer *et al.*^1^ evaluated the use of pericardial late gadolinium enhancement (LGE) as a biomarker in pericarditis. Recurrent pericarditis (RP) presents therapeutic challenges, necessitating novel biomarkers for risk stratification. In the RHAPSODY trial substudy, pericardial LGE association with pericarditis recurrence time was assessed. RHAPSODY, a Phase 3 trial on RP, included patients with active recurrence eligible for a cardiac magnetic resonance (CMR) imaging substudy. Moderate/severe LGE (*n* = 16) correlated with higher recurrence rates and CRP levels compared to trace/mild LGE (*n* = 9). Placebo recipients with moderate/severe LGE had shorter median recurrence time (4.2 weeks) than those with trace/mild LGE (10.7 weeks). Preliminary findings suggest pericardial LGE as a potential imaging biomarker for treatment duration and recurrence risk in RP, warranting larger studies to effectively validate LGE as a valuable prognostic tool in managing RP.

Abdula *et al.*^2^ evaluated the use of accelerated CMR acquisition for pulmonary artery pressure estimation. Non-invasive estimation of mean pulmonary artery pressure (mPAP) using CMR is a promising approach. This study introduces a compressed sensing (CS) accelerated CMR acquisition technique, significantly reducing scan times while preserving accuracy. Fifty-one patients undergoing clinical CMR at 1.5 T or 3 T were scanned using both CS-accelerated and non-CS-accelerated flow sequences. Analysis of pulmonary artery (PA) vortex duration estimated mPAP. CS-accelerated and non-CS-accelerated methods yielded comparable mPAP estimates, with a mean bias of 0.1 ± 1.9 mmHg and an intraclass correlation coefficient of 0.97 (95% confidence interval 0.94–0.98). CS-accelerated acquisition significantly reduced scan time by 79% (1 min 55 s ± 27 s vs. 9 min 6 s ± 2 min 20 s, *P* < 0.001). By enhancing clinical utility, this innovation facilitates the broader adoption of CMR in assessing pulmonary haemodynamics, with potential implications for various pulmonary vascular disorders.

Arvidsson *et al.*^3^ performed a non-invasive computation of left ventricular (LV) pressure–volume (PV) loops. LV PV loops, the gold standard for physiological insight, often necessitate invasive measurements of ventricular pressure, limiting their clinical and research utility. In this preliminary study, CMR and brachial cuff blood pressure for PV loop computation. Evaluation against invasive LV pressures in four heart failure (HF) patients revealed high fidelity, with strong correlation and minimal bias for various parameters including stroke work, potential energy, and ventricular efficiency. This approach enhances our understanding of ventricular mechanics and may inform clinical decision-making in HF management.

HF remains one of the most important topics in cardiovascular disease, as demonstrated by these two papers. Systolic ejection time (SET) is a treatment target in HF with reduced left ventricular ejection fraction. Morbach *et al.*^4^ established reference values for heart rate-corrected SET (SETc) and assessed its prognostic value in decompensated HF. In the population-based STAAB study (4836 participants, mean age 55 ± 12 years, 52% women), mean SETc was 328 ± 18 ms, increasing with age (+4.7 ms per decade), was shorter in men and correlating with arterial elastance (*r* = 0.30; all *P* < 0.001). Hospitalized HF patients showed shorter SETc, with differences among HF subtypes. Proportional hazard regression revealed that in-hospital SETc prolongation predicted favourable outcomes in HFrEF (age- and sex-adjusted hazard ratio: 0.38; 95% confidence interval: 0.18–0.79) but adverse outcomes in HFpEF (hazard ratio: 2.39; 95% confidence interval: 1.24–4.64). These findings suggest a tailored treatment approach in HF patients based on individualized SETc assessment.

The other paper on HF published by Simon *et al.*^5^ conducted on middle-aged UK Biobank participants without overt cardiovascular disease, aimed to explore the association between increased carotid intima-media thickness (IMT) and cardiac structure/function changes. In 4301 individuals (mean age 61.6 ± 7.5 years, 45.9% male), increasing IMT quartiles correlated with enlarged LV and right ventricular (RV) and left atrial volumes, higher LV mass, and decreased LV end-systolic circumferential strain, torsion, and atrial ejection fractions (all *P* < 0.05). These associations remained significant after adjustment for multiple confounders. The authors concluded that elevated carotid IMT independently associates with cardiac chamber enlargement, increased LV mass, and subtle LV dysfunction, indicating a potential for risk stratification and early detection of cardiac changes beyond traditional risk factors.

Wu *et al*.^6^ performed a large-scale investigation on ascending aortic diameter in Asian populations that are lacking, as well as evidence on the distribution of hypertension (HP), bicuspid aortic valve (BAV), and Marfan syndrome (MFS). This study fills this gap by examining a vast dataset of individuals undergoing cardiac ultrasound in China. Data from 698 795 individuals undergoing cardiac ultrasound were retrospectively analysed, with 647 087 included. This study reveals age- and gender-related trends in ascending aortic diameter and elucidates the prevalence of aortic dilation, aneurysm, and dissection in hypertensive, BAV, and MFS populations. In the normal population, mean ascending aortic diameter was 28.1 ± 3.2 mm, increasing with age (*P* < 0.001). Prevalence of aortic dilation, aneurysm, and dissection in HP individuals was 12.83%, 2.70%, and 4.77%, respectively. MFS individuals showed rates of 43.92%, 35.31%, and 26.11%, respectively. BAV patients had high incidences of aortic dilation (37.00%) and aneurysm (16.46%), with low aortic dissection incidence (0.74%). Aortic dissection mainly occurred at diameters <55 mm. Overall, dissection incidence increased with aortic diameter, revealing an ‘aortic paradox’. The findings shed light on the distribution of aortic dimensions and underscore the significance of early detection and management of aortic pathologies in these populations.

In this last paper, Alsharqui *et al.*^7^ used a Machine Learning approach for Cardiac Remodelling Assessment in Hypertension. In this paper, a novel semi-supervised machine learning approach was used to develop a summary score for cardiac remodelling in HP, aiming for early detection before significant left ventricular hypertrophy occurs. Using data from three UK studies on young adults, a contrastive trajectories inference method identified variance in 66 echocardiography variables between hypertensive and normotensive groups. A normalized score, indicating the extent of cardiac remodelling, was derived for each individual. Model stability and clinical interpretability were confirmed, with the score showing expected HP-related patterns and responsiveness to a 16-week exercise intervention. This quantitative score offers the potential for personalized prevention advice, pending further clinical validation.

## Review papers

In addition to the original articles described above, *EHJ-IMP* has seen remarkable engagement with reviews addressing diverse topics. Notably, studies on cardiopulmonary exercise testing (CPET), cardiac conduction devices (CCD), temporary mechanical circulatory support (tMCS), and diagnostic performance in left ventricular thrombus (LVT) detection have garnered significant attention from clinicians and researchers. These contributions underscore the journal's commitment to advancing knowledge across the spectrum of cardiovascular medicine.

Del Punta *et al.*^8^ evaluated an integrated approach to exercise intolerance that poses a significant challenge in various cardiovascular conditions. This article delves into the combined use of CPET and exercise-stress echocardiography to comprehensively phenotype the mechanisms underlying exercise intolerance. By refining diagnostic strategies and enhancing risk stratification, this integrated approach holds promise in guiding therapeutic interventions tailored to individual patient needs.

Tun *et al.*^9^ performed an update on the evaluation of CCD on chest radiographs. With the rising complexity of cardiac conduction device insertions, this review provides a comprehensive overview of assessing CCDs on chest radiographs. Addressing internal medicine residents and cardiologists, it elucidates the appearances of various CCDs and outlines a step-by-step guide for assessing device position and associated complications. This resource proves invaluable for clinicians involved in managing patients with CCDs, aiding in both immediate post-insertion evaluation and outpatient follow-up care.

In their review, Fortuni *et al.*^10^ described the Management of Temporary Mechanical Circulatory Support Devices. tMCS devices play a critical role in managing cardiogenic shock and high-risk procedures. This review discusses the diverse range of tMCS devices, emphasizing the importance of invasive and non-invasive haemodynamic parameters in device selection, initiation, and monitoring. By providing insights into the advantages and drawbacks of different tMCS modalities, this review aids clinicians in optimizing patient outcomes in various clinical scenarios.

Phuah *et al.*^11^ conducted a systematic review and meta-analysis to compare the diagnostic performance of transthoracic echocardiography (TTE) in LVT. LVT detection poses diagnostic challenges, with TTE being a commonly used modality. This systematic review and meta-analysis compare the diagnostic performance of TTE against CMR, the gold standard investigation for LVT. Studies involving 2113 patients undergoing both TTE and CMR were analysed. Non-contrast TTE had sensitivity and specificity of 47% and 98%, respectively, while contrast TTE had 58% sensitivity and 98% specificity. Apical wall motion scoring on non-contrast TTE had 100% sensitivity and 54% specificity. Despite high specificity, TTE's sensitivity for LVT detection varies across different techniques, highlighting the need for judicious utilization and consideration of additional imaging modalities, such as CMR, in cases of diagnostic uncertainty. The study provides valuable insights into optimizing diagnostic strategies for LVT detection, thereby guiding clinical practice and informing future research directions.

Last but not least, Lakshmanan and Mbanze^12^ published a paper that incorporates two of the pillars of *EHJ-IMP*: collaboration and inclusivity. Cardiovascular diseases are the leading global cause of morbidity and mortality, with disparities between high-income and low- to middle-income countries. Non-invasive cardiac imaging plays a crucial role in diagnosis and management but access inequalities persist due to financial constraints and healthcare disparities. A survey by the International Atomic Energy Agency revealed a global reduction of 64% in diagnostic procedure volumes from March 2019 to April 2020, with varying recovery rates. Recovery was robust in high-income countries (108%) but less so in low-income countries in Africa (30%), indicating a potentially significant collateral impact on cardiovascular outcomes. This review underscores the urgent need for sustainable solutions to ensure equitable access to diagnostic cardiovascular imaging worldwide.

## Conclusion

In summary, the compilation of research and review papers presented in *EHJ-IMP* underscores the significant strides made in the application of imaging modalities in cardiovascular medicine. Each featured paper represents a critical contribution to the field, shedding light on novel techniques, diagnostic approaches, and interventions. As we reflect on the achievements of these top papers, we are reminded of the enduring impact of collaborative scientific endeavours in shaping the future of cardiovascular care.

## Lead author biography

Dr Alessia Gimelli is a cardiologist and nuclear medicine specialist. She is the Director of the Health Quality System at Fondazione Toscana G. Monasterio, Pisa, Italy. Dr Gimelli has deep experience in clinical research on cardiovascular diseases and cardiovascular imaging and has contributed to several international high-profile projects. She is the Editor-in-Chief of the *European Heart Journal—Imaging Methods and Practice Journal*.
